# Retinoic acid influences the timing and scaling of avian wing development

**DOI:** 10.1016/j.celrep.2021.110288

**Published:** 2022-01-25

**Authors:** Holly Stainton, Matthew Towers

**Affiliations:** 1 School of Biosciences, University of Sheffield, Western Bank, Sheffield S10 2TN, UK

**Keywords:** timing, chick, quail, patterning, limb development, retinoic acid, growth, Shh, scaling

## Abstract

A fundamental question in biology is how embryonic development is timed between different species. To address this problem, we compared wing development in the quail and the larger chick. We reveal that pattern formation is faster in the quail as determined by the earlier activation of 5′*Hox* genes, termination of developmental organizers (*Shh* and *Fgf8*), and the laying down of the skeleton (*Sox9*). Using interspecies tissue grafts, we show that developmental timing can be reset during a critical window of retinoic acid signaling. Accordingly, extending the duration of retinoic acid signaling switches developmental timing between the quail and the chick and the chick and the larger turkey. However, the incremental growth rate is comparable between all three species, suggesting that the pace of development primarily governs differences in the expansion of the skeletal pattern. The widespread distribution of retinoic acid could coordinate developmental timing throughout the embryo.

## Highlights

• Quail wings develop faster than chick and turkey wings
• Retinoic acid can set the species timing of wing development
• Developmental timing is independent of growth and scales the skeletal pattern

## Graphical abstract

**Figure:**
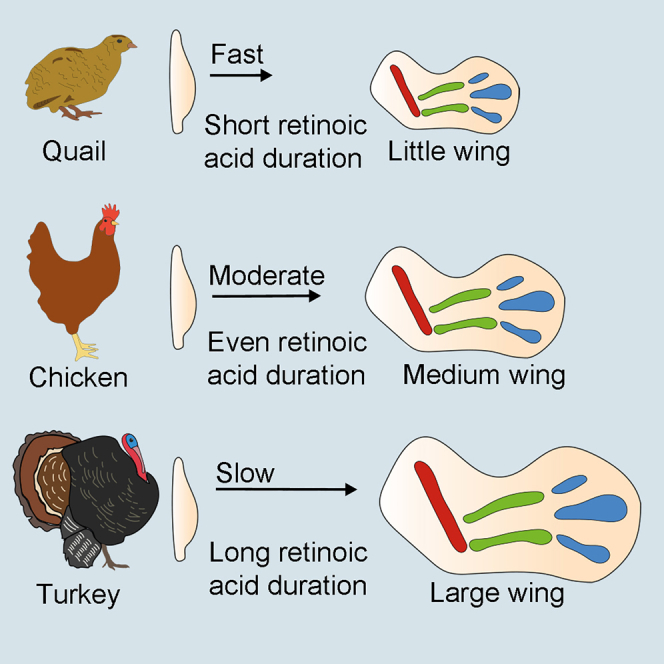

## Teaser

Time determines the pace of development. Stainton and Towers show that the duration of retinoic acid signaling sets the timing of avian wing development and influences skeletal pattern scaling. Retinoic acid is used throughout the embryo, and it could play a general role in developmental timing and size determination.

## Introduction

Developmental timing can be defined as the pace at which embryos progress through a series of morphological states and sequential patterning events. Although we know much about how the embryo develops, our knowledge about timing in different-size species is not currently well understood (Ebisuya and Briscoe, 2018; Rayon and Briscoe, 2021). Recent *in vitro* approaches have revealed that the rate of protein degradation in embryonic mouse cells is approximately twice as fast as that found in human cells, and that this correlates well with the pace of somitogenesis and motor neuron differentiation (Matsuda et al., 2020; Rayon et al., 2020). The avian wing provides an excellent *in vivo* system with which to understand species developmental timing during embryogenesis, as we possess in-depth knowledge of the underlying mechanisms that pattern the proximodistal axis (humerus to digits), which rely on the integration of extrinsic signaling and autonomous timing processes (McQueen and Towers, 2020). The specification of the chick wing skeletal pattern involves a switch from proximal signaling from the body wall (humerus/stylopod specification) to an autonomous timing mechanism operating in mesoderm cells at the distal tip of the outgrowing bud (digit/autopod specification) (Cooper et al., 2011; Rosello-Diez et al., 2011, 2014; Saiz-Lopez et al., 2015, 2017; Pickering et al., 2018). Recent evidence suggests that the transition from proximal signaling to autonomous timing occurs during forearm/zeugopod specification (Rosello-Diez et al., 2014; Saiz-Lopez et al., 2015; Delgado et al., 2020). Retinoic acid (RA) emanating from the trunk of the embryo is implicated as the extrinsic signal involved in proximal specification (Mercader et al., 2000; Mic et al., 2004; Cooper et al., 2011; Rosello-Diez et al., 2011). The specification of positional values that encode the different segments of the limb is associated with the progressive expression of genes encoding 5′ Hox proteins: *Hoxa/d10* provide a readout of stylopod specification, and then *Hoxa/d11* followed by *Hoxa/d13* provide readouts of zeugopod and autopod specification, respectively (Nelson et al., 1996; Tabin and Wolpert, 2007).

Following proximodistal specification, complex reciprocal epithelial-mesodermal signaling interactions sustain limb outgrowth as the population of *Sox9*-expressing prechondrogenic cells expand and lay down the skeletal pattern. The undifferentiated distal mesoderm produces a signal encoded by the Bmp inhibitor, Gremlin1, which maintains the overlying apical ectodermal ridge (Zuniga et al., 1999; Khokha et al., 2003). This structure is a thickening of the distal epithelium that maintains limb outgrowth (Saunders, 1948; Summerbell et al., 1973) and is marked by the expression of *Fgf8* (Crossley and Martin, 1995). However, the duration of proliferative growth of the chick wing is an autonomous property of the undifferentiated mesoderm and is controlled by the progressive Bmp-dependent decline in G1-S-phase entry in both the distal tip and in the polarizing region (Pickering et al., 2018, 2019)—a region of posterior-distal mesoderm that produces Sonic hedgehog (Shh)—the secreted signal that specifies positional values along the antero-posterior axis (thumb to little finger) (Riddle et al., 1993; Tickle and Towers, 2017). The laying down of the skeletal pattern along the proximodistal axis is complete when *Sox9* is expressed in all condensing cartilage cells, proliferative outgrowth terminates in the distal mesoderm, and the apical ectodermal ridge regresses (Pickering et al., 2018). However, it is unknown how the timing of proximodistal patterning is controlled in different-size species.

Here, we demonstrate that proximodistal developmental timing is accelerated in smaller avian species and is associated with a quicker rate of *5′Hox* gene activation and the earlier laying down of the skeletal pattern. We implicate RA as the signal that sets the pace of development, and we reveal that it is sufficient to switch quail to chick and chick to turkey timing. We show that the timing of development alongside a comparable growth rate results in species differences in the expansion and scaling of the skeletal pattern.

## Results

### Proximodistal patterning timing in quail and chick wings

**Figure 1:**
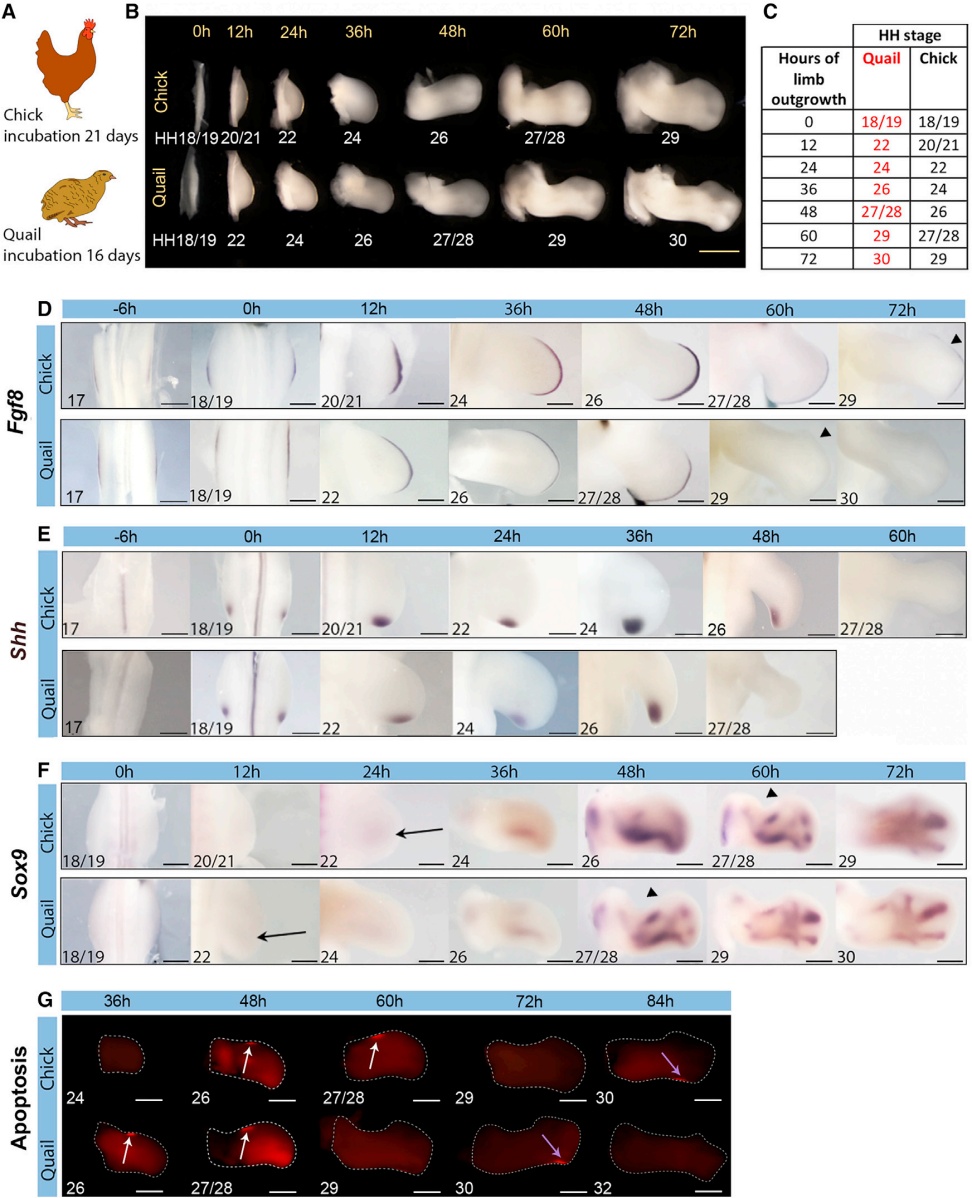
Proximodistal patterning timing in quail and chick wings (A) Schematics of the chick and quail that have 21- and 16-day incubation periods, respectively. (B and C) Hamburger Hamilton (HH) staging of chick and quail wings over 72 h from HH18/19 until HH29 and HH30, respectively; 0 h refers to day 3 of incubation. (D) *Fgf8* is initiated at the same time and is detectable until HH29: 72 h in the chick wing and 60 h in the quail wing, indicated by arrowheads. The HH stage is noted in the bottom left-hand corner. (E) *Shh* is initiated at the same time and is detectable until HH26: 48 h in the chick wing and 36 h in the quail wing. (F) *Sox9* expression is advanced by 12 h in the quail wing compared to the chick (compare 48-h quail wing and 60-h chick wing, arrowheads). Black arrows indicate the onset of *Sox9* expression at HH22. At least 10 embryos were analyzed at each stage for determining gene expression. (G) Anterior (white arrows) and posterior (purple arrows) necrotic zones (red) are 12 h advanced in quail wings compared to chick wings (n = 9–12 for each stage). Scale bars: (A) 1 mm; (D) 12 h, 200μm; 36 h, 400μm; 6 h, 0 h, 48 h, and 60 h, 500μm; 72 h, 600μm; (E) 12 h and 24 h, 300μm; 36 h, 500μm; 6 h, 0 h, 48 h, and 60 h, 500μm; 48 h quail, 700μm; (F) 12 h, 24 h, 250μm; 0 h, 400μm; 36 h, 500μm; 48 h and 60 h, 600μm; 72 h, 700μm; (G) 36 h, 48 h, and 60 h, 500μm; 72 h, 600μm; 84 h −750μm.

To understand how development is timed between differently sized species, we staged quail wings in reference to the Hamburger Hamilton (HH) staging system of the larger chick (Figures 1A–1C). The quail and chick are both in the Galliformes order and have incubation periods of 16 and 21 days, respectively. At day 3 of incubation, quail and chick embryos are at an equivalent stage (HH18/19), as determined by the appearance of the allantois (extra-embryonic membrane sac) and somites extending into the tail bud (∼36 pairs) (Hamburger and Hamilton, 1951; Padgett and Ivey, 1960; Ainsworth et al., 2010). At this stage, the chick embryo is slightly wider than the quail embryo (between the wing buds), but it is not significantly different in length (from the tip of the tail bud to the metencephalon; Figure S1). Assignment of the HH stage is based on the shape and gross morphology of the wing. Thus, at HH18/19, wing buds can be identified as slight symmetrical bulges protruding from the flank of the embryo (for the rest of the article, we define this as 0 h, the last time point at which quail and chick wings are at an equivalent stage of development; Figures 1B and 1C). The full pattern of skeletal elements is laid down by HH29, when the apical ectodermal ridge regresses, which the chick reaches at 72 h and the quailreaches at 60 h (Figures 1B and 1C). Correspondingly, *Fgf8* is activated at the same time and persists in the chick wing until 72 h and in the quail wing until 60 h (Figure 1D). In a similar manner, there is a 12-h difference in the timing of *Shh* expression in quail and chick wing polarizing regions. Thus, *Shh* transcription is activated at the same time and it persists until 36 h in the quail and until 48 h in the chick (until HH26 in both species; Figure 1E). Furthermore, there is a 12-h difference in the timing of *Sox9* expression, which is a marker of the expanding population of differentiating chondrogenic cells that prefigure the entire skeletal pattern by HH29 (Healy et al., 1999). Thus, the onset of *Sox9* expression occurs at HH22 in both species, and this 12-h difference in timing can be appreciated by its similar spatial pattern at HH27/28, which is reached at 48 h in the quail and 60 h in the chick (Figure 1F). To further assess the timing of development, we analyzed the anterior and posterior necrotic zones, which are regions of apoptosis in the wing (Saunders and Gasseling, 1962; Fernandez-Teran et al., 2006). As with differentiating chondrogenic cells, there is a 12-h difference in the timing of apoptosis between quail and chick wings. Thus, the anterior necrotic zone persists between HH26 and HH27/28 and the posterior necrotic zone becomes visible at HH30 in both species (Figure 1G). These observations reveal that the developmental progression from HH18/19 to HH22 is faster in the quail wing bud, which results in patterning being completed 12 h earlier than it is in the chick wing (HH29 in both species).

### Relationship between growth and proliferation in quail and chick wings

**Figure 2:**
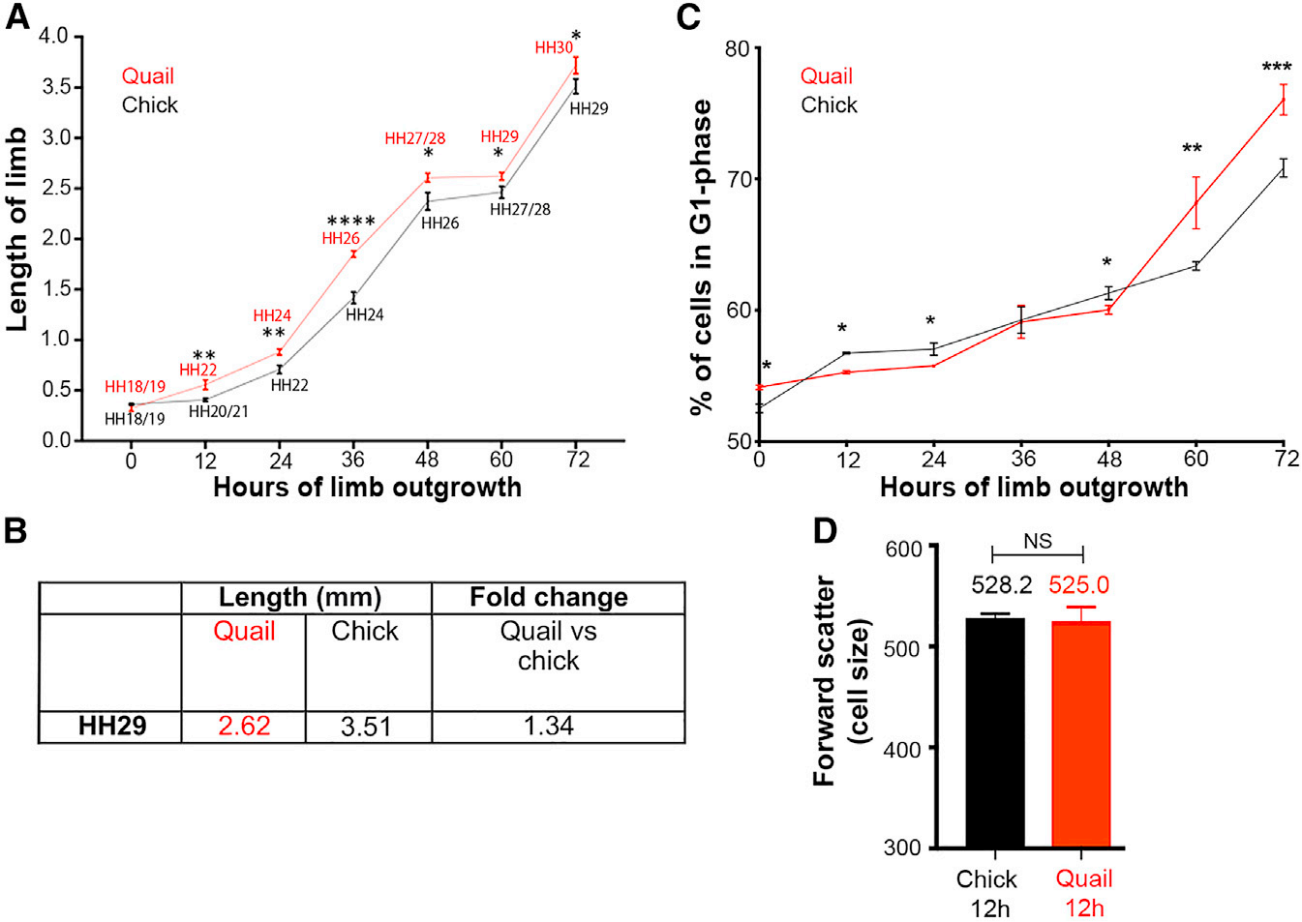
Growth and proliferation in quail and chick wings (A) Proximodistal lengths of quail and chick wing buds (body wall to tip of wing bud) are not significantly different at 0 h, but are significantly different between 12 and 72 h, indicated by Student’s t test (12-h p = 0.0017, 24-h p = 0.0079, 36-h p ≤ 0.0001, 48-h p = 0.0382, 60-h p = 0.0280, 72-h p = 0.0268). Quail wings grow at a significantly faster rate between 0 and 12 h, as determined by Student's t test (p = 0.0462) (n = 4). However, there is no significant difference in incremental changes in growth rates from 12 to 72 h as determined by Wilcoxon tests (p = 0.688 (n = 4–14). (B) Average lengths of quail and chick wings and fold differences atHH29. (C) The proportion of distal mesoderm cells in G1 phase indicates that the quail has a significantly faster cell cycle at 12 h (p = 0.0165) 24h (p = 0.0144) and 48 h (p = 0.0175), and the chick at 0 h (p = 0.0123), 60 h (p = 0.0018), and 72 h (p = 0.0004). Student’s t tests were performed on n = 3 repeats of 8–12 pooled blocks of distal mesenchyme. (D) Cell size is equivalent in quail and chick wing buds at 12 h (forward scatter of the signal is shown as arbitrary units). Student’s t tests were performed on n = 3 repeats of 6–10 pooled blocks of distal mesenchyme. ^∗^p ≤ 0.05, ^∗∗^p ≤ 0.01 ^∗∗∗^p ≤ 0.001 ^∗∗∗∗^p ≤ 0.0001.

We determined whether the 12-h difference in developmental timing between quail and chick wings is associated with proximodistal growth by measuring their lengths from the trunk to the distal tip. Between 0 and 12 h, quail wing buds grow at a significantly faster rate than chick wing buds and therefore become significantly longer (Figure 2A). However, after 12 h, the incremental growth rates of quail and chick wings are not significantly different up until the last time point measured at 72 h (Figure 2A). Therefore, the developmental progression through HH stages becomes uncoupled from the comparable rate of incremental growth. For instance, growth plateaus between 48 and 60 h in both species, which is between different HH stages (HH27/28-HH29 in quail and HH26-HH27/28 in chick; Figure 2A). The asynchrony of developmental timing and growth means that at HH29 (chick 72 h, quail 60 h; Figure 1C), the skeletal pattern is of a different size (1.34-fold longer in the chick compared to the quail; Figure 2B).

To determine whether changes in the rate of proliferation could account for the early difference in the growth rates of quail and chick wings, we used flow cytometric analyses to determine the proportion of distal mesoderm cells in G1 phase of the cell cycle (Saiz-Lopez et al., 2015; Pickering et al., 2018). Although most cells in the early wing bud are dividing, the percentage of G1 phase cells in the undifferentiated distal mesoderm provides an accurate indicator of the diminishing rate of proliferation during the patterning phase (Saiz-Lopez et al., 2015; Pickering et al., 2018). Between 0 and 12 h, a significantly faster rate of proliferation is maintained in the quail wing bud, and this could contribute to increased proximodistal elongation compared to the chick wing bud (Figure 2C). This conclusion is supported by the observation that cell size, as determined by flow cytometric analyses (forward scatter measuring fluorescence as arbitrary units; Collier, 2000), is not significantly different between the two species at 12 h (Figure 2D). After 12 h, significant changes in proliferation rates correlate better with developmental timing than with growth. This can be appreciated by the acute increase in the percentage of G1 phase cells (indicative of a decreasing proliferation rate) at HH27/28 in both species (Figure 2D; 48 h in the quail and 60 h in the chick). Therefore, between 0 and 12 h, proliferation could account for the slightly enhanced growth rate of quail wing bud. However, at later stages, proliferation is associated with developmental timing and is unlikely to account for major differences in the rate of proximodistal elongation between species.

### Proximodistal specification timing in quail and chick wings

**Figure 3:**
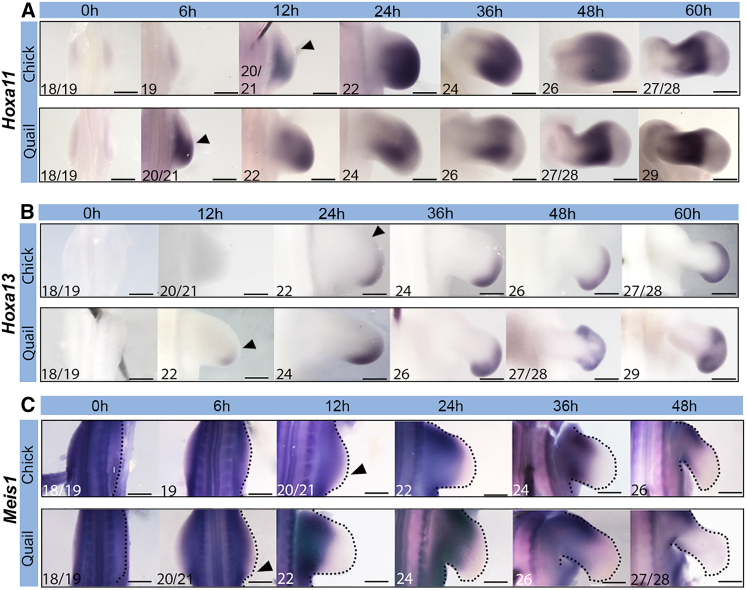
Proximodistal specification timing in quail and chick wings (A) *Hoxa11* is expressed at HH20/21, which is 6 h earlier in the quail compared to the chick; arrowheads indicate onset of expression. HH stage noted in the bottom left corner. (B) *Hoxa13* is expressed at HH22, which is 12 h earlier in the quail compared to the chick; arrowheads indicate onset of expression. (C) *Meis1* expression is downregulated in the distal part of the wing at HH20/21, which is 6 h earlier in the quail compared to the chick, as indicated by arrowheads. Scale bars: (A) 6 h, 12 h, and 24 h, 300μm; 0 h and 36 h, 500μm; 48 h and 60 h, 600μm; (B) 12 h and 24 h, 200μm; 0 h and 48 h chick, 300μm; 36 h, 400μm, 48 h quail, 600μm, 60 h, 800μm; (C) 12 h and 24 h, 300μm; 0 h and 6h, 400μm; 36 h, 500μm; 48 h, 650μm.

We addressed whether the 12-h difference in developmental timing between the quail and chick is associated with the pace of proximodistal positional value specification. The switch from proximal specification (stylopod) to intermediate specification (zeugopod) is indicated by the activation of *Hoxa11* expression, and the switch from intermediate specification (zeugopod) to distal specification (autopod), by the activation of *Hoxa13* expression (Nelson et al., 1996; Saiz-Lopez et al., 2015; Delgado et al., 2020). The expression of *Hoxa11* is detectable at 6 h in the quail wing bud and at 12 h in the chick (Figure 3A; HH20/21 in both cases), which resolves into a 12-h difference in timing at later stages (compare 48-h quail to 60-h chick). By contrast, the expression of *Hoxa13* is detectable at 12 h in the quail wing bud and at 24 h in the chick (Figure 3B; HH22 in both cases), and this 12-h difference in timing is maintained throughout outgrowth. In addition, the appearance of a *Meis1-*free domain in the distal part of the limb is also an indicator of autopod specification, and provides a readout of proximal RA signaling from the flank of the embryo (Mercader et al., 2000). The loss of distal *Meis1* expression occurs at ∼6 h in the quail wing bud and at 12 h in the chick (Figure 3C; HH20/21 in both species), and as with *Hoxa11*/*13* timing, resolves into a 12-h difference at later stages. Consistent with this observation, the expression levels of the gene encoding the RA-degrading enzyme Cyp26b1 rise at a faster rate in the quail wing bud compared to the chick (Figure S2). Thus, the transition from stylopod to zeugopod specification occurs 6 h later in the chick wing bud compared to the quail, and the transition from zeugopod to autopod specification occurs 12 h later.

### Stability and resetting of quail and chick wing developmental timing

To gain insights into how the 12-h difference in developmental timing is set in quail and chick wings, we performed a series of reciprocal tissue grafting experiments to ascertain whether it is reset or maintained. We chose the polarizing region, as it expresses *Shh* and regulates its cell cycle parameters for an autonomously timed duration in the chick wing (Figure 1E) (Chinnaiya et al., 2014; Pickering et al., 2019).

**Figure 4:**
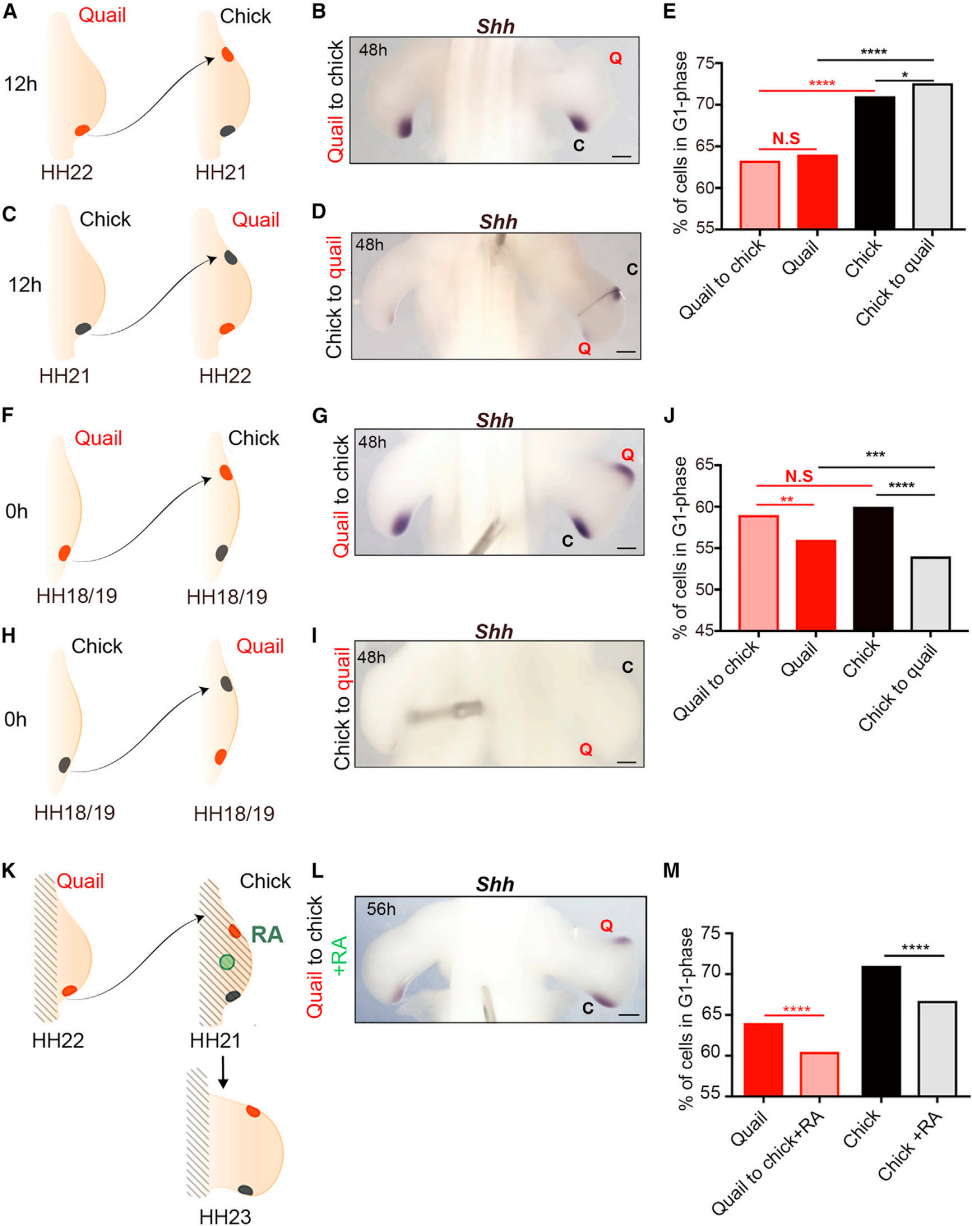
Resetting potential of species developmental timing in polarizing region grafts (A–D) In interspecies polarizing region grafts between 12-h quail (HH22) and chick (HH21) wings (made to the anterior margins of host wing buds), *Shh* expression is maintained according to donor timing (B, n = 7/10, D, n = 5/7). (E) The percentage of cells in G1 phase of the cell cycle in the grafts is close to donor values, but are significantly different from the host polarizing region 24 h after the graft was performed (Pearson’s χ^2^ test). p values: quail versus quail grafted to chick = 0.24, chick versus quail grafted to chick = 0.00001, chick versus chick grafted to quail = 0.013, and quail versus chick grafted to quail = 0.00001. (F–I) In interspecies polarizing region grafts between 0-h quail and chick wings (HH18/19 in both species), *Shh* expression is reset according to host timing (G, *n* = 9/10, I, n = 3/5). (J) The percentage of cells in G1 phase of the cell cycle in the grafts is reset close to host values 24 h after the graft was performed (Pearson’s χ^2^ test) p values: quail versus quail grafted to chick = 0.02, chick versus quail grafted to chick = 0.09, chick versus chick grafted to quail = 0.00001, and quail versus chick grafted to quail = 0.00012. (K and L) HH19 chick wings were treated with retinoic acid (RA)-soaked beads (green circle), and at 12 h (HH21), they received polarizing regions grafts from 12-h quail wings (HH22); black hatch marks indicate presumed RA distribution from the bead, *Shh* expression is prolonged until ∼56 h in both the host and donor (L, n = 3/4). (M) The percentage of cells in G1 phase of the cell cycle in the RA-treated quail and host chick wing polarizing region is significantly reduced compared to control polarizing regions at 24 h after grafting (Pearson's χ^2^ test). N.S. *= >*0.05, ^∗∗∗^p ≤ 0.001, ^∗∗∗∗^p ≤ 0.0001. Scale bars: 500μm.

We performed interspecies polarizing region grafts to the anterior margins of host wing buds at 12 h when developmental timing is advanced in the quail compared to chick (HH21 chick and HH22 quail; Figures 4A and 4C; note that stage-matched intraspecies control grafts maintain their normal duration of *Shh* expression [Figure S3]). We found that grafts performed at these stages maintain their species timing of *Shh* expression. Thus, at 48 h, *Shh* expression is undetectable in quail cells grafted to a chick wing, but it is detectable in the host (Figure 4B). In the reciprocal experiment, *Shh* expression is detectable in chick cells grafted to a quail wing, but it is undetectable in the host (Figure 4D). In addition, both donor quail and chick wing bud polarizing region cells maintain species-specific cell cycle parameters typical of their donor age 24 h after the grafts were performed (Figure 4E; 63.3% G1 phase cells in quail graft versus 71% in chick host; 72.6 chick graft versus 64% in quail host). Rather than being an autonomous process, it was originally suggested that the termination of *Shh* expression requires the displacement of *Gremlin1-*expressing cells by a critical distance from the polarizing region to break down a self-propagating extrinsic signaling loop (Scherz et al., 2004). However, in chick wings that received quail polarizing region grafts, *Shh* expression is terminated in donor cells independently of their proximity to the duplicate domain of *Gremlin1* expression in the host (Figures S4A and S4B). Thus, these findings show that the timing of both *Shh* expression and cell cycle parameters are autonomously determined after HH21.

We also performed interspecies polarizing region grafts at 0 h, when developmental timing is equivalent in chick and quail wing buds (HH18/19; Figures 4F and 4H). Unlike the 12-h grafts, *Shh* expression is reset to host timing in 0-h interspecies grafts. Thus, at the 48-h time point, *Shh* expression duration is prolonged in quail cells in a chick wing, and it is prematurely terminated in chick cells in a quail wing (Figures 4G and 4I, respectively; note that 0-h intraspecies control grafts maintain their normal duration of *Shh* expression; Figure S3). In addition, cell cycle parameters are also reset close to host values in both donor quail and chick wing bud polarizing regions 24 h after grafts were performed (Figure 4J; 59% G1 phase cells in quail graft versus 60% in chick host; 54% in chick graft versus 56% in quail host). Furthermore, the species timing of *Shh* can be reset in HH22 interspecies polarizing regions grafted to an earlier HH18/19 host wing bud, which is consistent with our previous findings on intraspecies grafts (Chinnaiya et al., 2014) (Figures S4C and S4D).

These results reveal that both *Shh* expression and cell cycle parameters are autonomously maintained in 12-h polarizing region grafts when developmental timing is offset between quail and chick wings (HH21 versus HH22), but that they can be reset in 0-h grafts when developmental timing is equivalent (HH18/19).

### RA can reset developmental timing

The ability of the host environment to reset developmental timing before 12 h coincides with high RA signaling in the distal part of the limb, as indicated by *Meis1* expression (Figure 3C). Therefore, we asked whether the transient maintenance of RA signaling by carrier beads for 12 to 20 h in the host chick wing bud (Eichele et al., 1984, 1985) would reverse the autonomy of *Shh* expression timing in quail polarizing region grafts made at the 12-h time point (Figure 4K). By transiently prolonging RA signaling, *Shh* expression timing is reset in the quail polarizing region graft, as it is maintained for approximately the same duration as it is expressed in the host chick polarizing region (∼56 h, Figure 4L; compare with failure to reset timing in the same experiment minus RA, Figures 4B and 4D). It is worth noting that the duration of *Shh* expression is also extended in the RA-treated host chick polarizing region (compare to level of residual *Shh* expression in the contralateral untreated wing in Figure 4L). In addition, both donor quail and host chick polarizing regions in RA-treated wing buds have a significantly faster rate of proliferation 24 h after grafts were performed, as indicated by a lower percentage of cells in G1 phase compared to control untreated polarizing regions, which is consistent with an earlier stage of development (Chinnaiya et al., 2014) (Figure 4M; 60.5% G1 phase cells in quail graft versus 64% in control quails; 66.7% in RA-treated chick host versus 71% in control chicks). Therefore, developmental timing can be reset by transiently prolonging retinoic signaling.

### RA can set developmental timing

**Figure 5:**
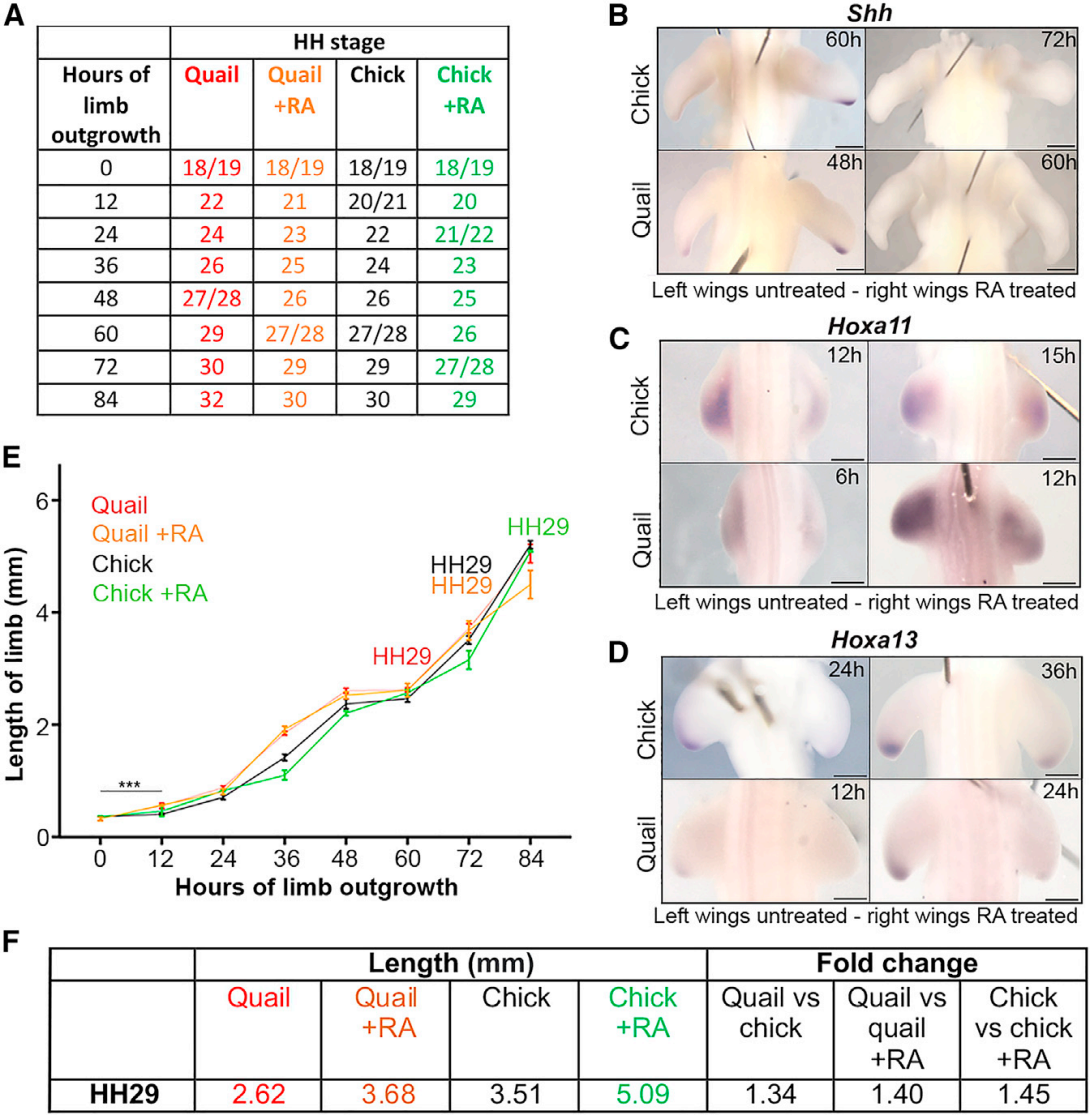
RA can set species developmental timing (A) HH stages of chick and quail wing buds treated with RA at 0 h, compared with the contralateral untreated wing. (B) RA-treated wings express *Shh* until 60 h in the chick (n = 4/4) and 48 h in the quail (n = 3/3), compared to 48 and 36 h in control untreated wings. (C) RA-treated wings express *Hoxa11* at 15 h in the chick (n = 3/5), and 12 h in the quail (n = 3/5), compared to 12 and 6 h in control untreated wings. (D) RA-treated wings express *Hoxa13* at 36 h in the chick (n = 3/4) and 24 h in the quail (*n* = 3/5), compared to 24 and 12 h in control untreated wings. (E) Quail wings grow at a significantly faster rate compared to chick wings between 0 and 12 h, as determined by Wilcoxon tests (^∗∗∗^p = 0.0008) (n = 7). However, there is no significant difference in incremental changes in growth rates from 12 to 72 h (p = 0.688; n = 6–14). After 12 h, Wilcoxon tests also reveal no significant difference in incremental growth rates between chick versus chick + RA, chick versus quail + RA, chick + RA versus quail, quail versus quail + RA, and chick + RA versus quail + RA (p = >0.99, >0.99, 0.563, 0.438, and 0.688, respectively); n = 4–16. (F) Lengths of quail, RA-treated quail, chick and RA-treated chick wings, and fold differences at HH29. Scale bars: 250μm.

The observation that RA can reset the species duration of *Shh* and maintain a faster proliferation rate typical of younger wings could suggest that it plays a general role in setting development timing. Thus, when RA is applied on beads to HH18/19 quail and chick wing buds at 0 h to transiently maintain it for 12–20 h past its normal duration (Eichele et al., 1984, 1985), the subsequent developmental progression through HH stages occurs ∼12 h later than normal, such that HH29 is reached at 72 h and 84 h, respectively (Figures 5A, S5 and S6). Therefore, RA-treated quail wings and untreated chick wings have a similar timing of HH stage progression. Correspondingly, as predicted from the polarizing region grafting experiments (Figures 4A and 4B), RA extends the duration of *Shh* expression for 12 h in both quail and chick wings (until 48 h and 60 h, respectively; Figure 5B), which is HH26 in both species. In a similar manner, prolonged RA exposure also delays zeugopod specification (*Hoxa11*) for 3 h in the chick and for 6 h in the quail (Figure 5C, HH20/21) and delays autopod specification *(Hoxa13*) for 12 h in both species (Figure 5D, HH22). However, consistent with published data (Rosello-Diez et al., 2014), inhibiting RA signaling at HH18 and HH19 does not precociously activate *Hoxa13,* due to its expression also being controlled by an undefined timing mechanism (Figure S7). These results show that prolonged RA treatment slows the development of both quail and chick wings.

We determined whether the pace at which RA-treated quail and chick wings develop is associated with the rate of growth along the proximodistal axis. Analyses of the data reveal that after 12 h, the incremental growth rates are not significantly different between RA-treated and untreated wing buds (Figure 5E). Therefore, the rate of growth is not linked to the pace of development. An implication of this finding is that because the duration, but not the rate of growth, varies considerably between quail and chick wings and also those treated with RA, this significantly influences the length of the fully patterned wing at HH29. Hence, RA-treated quail wings are 1.4-fold longer than untreated quail wings, and similarly, RA-treated chick wings are 1.45-fold longer than untreated chick wings (Figure 5F). These observations demonstrate that the development and growth of RA-treated quail wings and untreated chick wings is comparable.

### Growth and developmental timing of turkey wings

**Figure 6:**
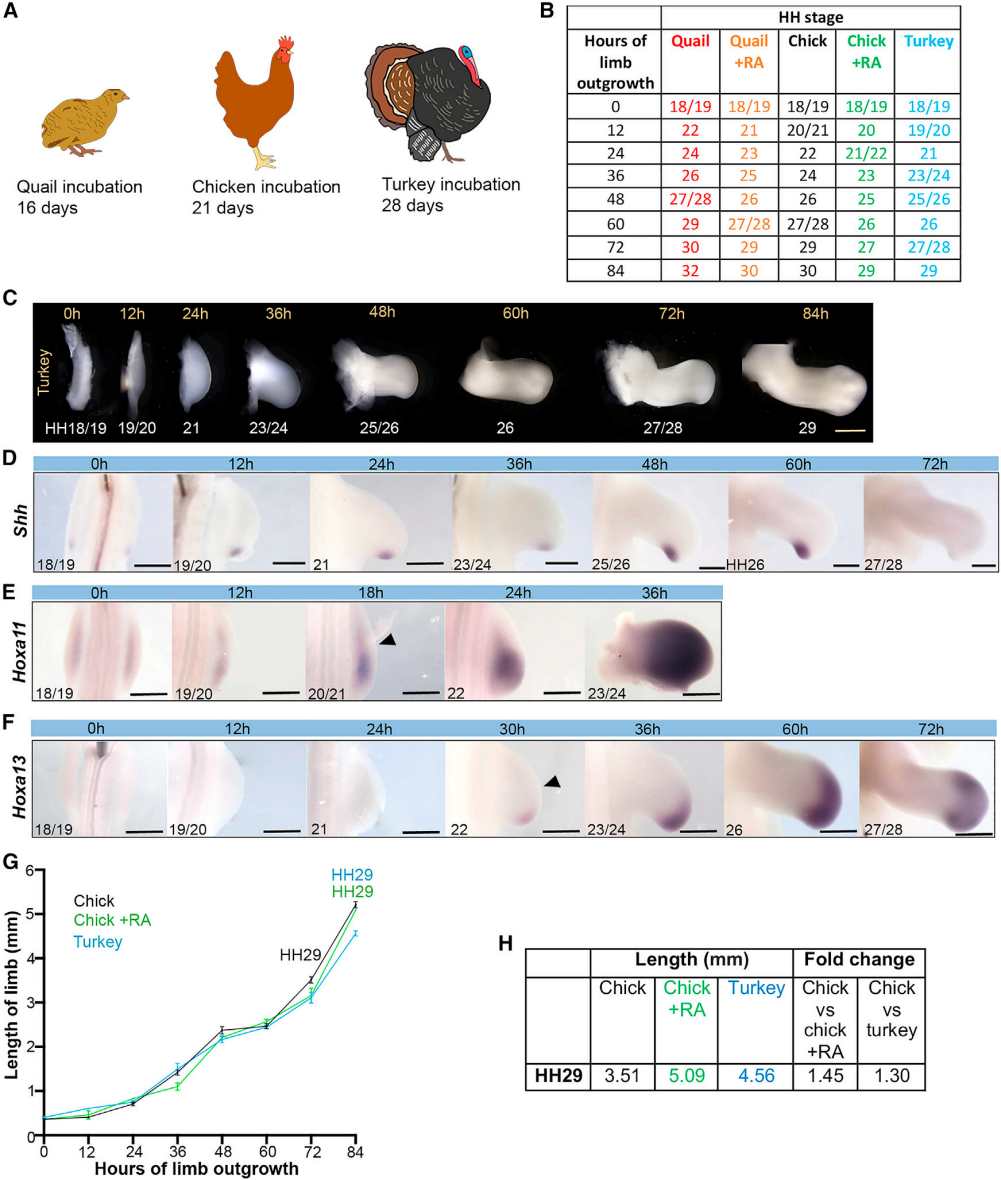
Developmental timing and growth of turkey wings (A) Schematics of quail, chick, and turkey that have 16-, 21-, and 28-day incubation periods, respectively. (B and C) HH staging of turkey wings over 84 h until HH29; 0 h refers to day 4 of incubation. (D) *Shh* is detectable until 60 h, which is HH26. HH stages noted in the bottom left of each panel. (E and F) *Hoxa11* is expressed at 18 h, which is HH20/21 (E) and (F) *Hoxa13* is expressed at 30 h, which is HH22; arrowheads indicate onset of expression. (G) Proximodistal lengths of chick, chick + RA, and turkey wing buds until 84 h (HH29 in turkey and chick + RA wings). Wilcoxon tests reveal no significant difference in incremental growth rates between turkey versus chick + RA and turkey versus chick (p = >0.437 and 0.219, respectively); n = 4–14. (H) Lengths of chick, RA-treated chick (chick + RA), and turkey wings and fold differences at HH29. Scale bars: (C) 750μm; (D) 500μm; (E) 0–36 h, 500μm; 60 h and 72 h, 600μm; (F) 0 h and 24 h, 400μm; 12 h and 36h–72h, 500μm.

We determined the timing of wing development in the turkey, which is a larger species than the chick, but also belongs to the Galliformes order and has an incubation period of 28 days (Figure 6A). We staged turkey wings according to the HH staging system of the chick, starting at HH18/19, which is reached at day 4 of incubation (note that the quail and chick reach HH18/19 at day 3) (Mun and Kosin, 1960). At HH18/19, the turkey embryo is significantly longer than the quail, but not the chick (from the tail bud to the metencephalon), and is similar in width to both quail and chick embryos (between the wing buds; Figures S8A and S8B). During the next 12 h, turkey wing buds progress to HH19/20, whereas chick wing buds reach HH20/21, and subsequently, the developmental timing of HH stage progression resolves into a 12-h difference between the two species by 48 h. Thus, HH29 is reached in 84 h in turkey wings compared to 72 h and 60 h for chick and quail wings, respectively (Figures 6B and 6C). In addition, *Shh* expression can be detected until 60 h (until HH26), *Hoxa11* expression (zeugopod specification) can be detected at 18 h (HH20/21) and *Hoxa13* expression (autopod specification) at 30 h (HH22) in turkey wings (Figures 6D–F). These timings are similar to those found in RA-treated chick wing buds (Figures 5B–5D).

Turkey wings also have an equivalent incremental rate of growth along the proximodistal axis when compared to untreated chick wings and RA-treated chick wings (Figure 6G). Therefore, since the duration but not the rate of growth varies considerably between chick wings and both chick wings treated with RA and turkey wings, this significantly influences the length of the fully patterned wing at HH29. Thus, turkey wings are 1.3-fold longer than chick wings and, similarly, RA-treated chick wings are 1.45-fold longer than untreated chick wings (Figure 6H). These results show that the development and growth of RA-treated chick wings and untreated turkey wings are comparable.

## Discussion

**Figure 7:**
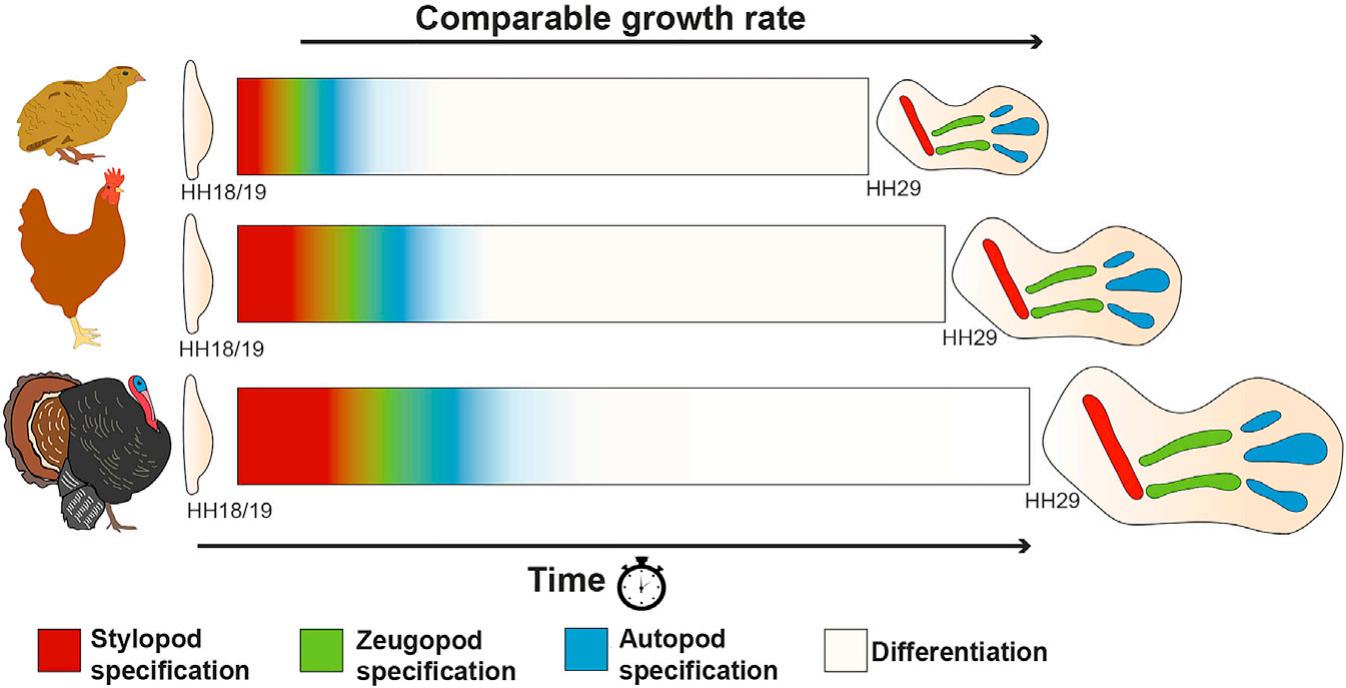
RA influences developmental timing and expansion of the avian wing skeletal pattern Schematics depicting the timing of proximodistal specification and differentiation in avian wings (quail, chick, and turkey) from HH18/19 until the end of the patterning phase at HH29, when the skeletal elements have been laid down. The stylopod (red) is specified when RA levels are high; the zeugopod (green) when RA levels are low , and the autopod (blue) by autonomous timing once RA has been removed. The duration of RA signaling and stylopod and zeugopod specification (red and green) varies between species; however, the duration of the autonomous program (blue and white) remains relatively constant. The timing of development (the pace) alongside a comparable growth rate results in species differences in the expansion of skeletal progenitor cells. Consequently, at HH29, when the complete skeletal pattern is laid down, there is a 1.34-fold difference in the size of quail and chick wings, a 1.3-fold difference in the size of chick and turkey wings, and a 1.75-fold difference in the size of quail and turkey wings (schematics of HH29 wings are scaled appropriately).

We have described a mechanism that can explain how the pace of embryonic wing development is controlled between different avian species (Figure 7). The duration of stylopod and zeugopod specification (red and green) is variable (12–30 h). Coinciding with the onset of autopod specification (blue), the autonomously timed program of distal development (white) then continues for a similar duration until patterning is complete and the skeletal elements have been laid down. However, because the rate of growth is equivalent between species, differences in developmental timing influence the expansion and scaling of the skeletal pattern (Figure 7). Our interspecies grafting experiments implicated RA as the signal that sets developmental timing. Transiently prolonging RA signaling slows down the rate of *5′Hox* gene activation, and therefore, chick and RA-treated quail wings develop comparably, as do turkey and RA-treated chick wings.

We provided insights into the underlying mechanism that determines the variable species duration of proximodistal specification (Figure 7). The degradation of a proximal signal, considered to be RA emanating from the flank of the embryo, influences the distribution of Meis1, the relative levels of which are proposed to permit the activation of 5′ *Hox* expression (Delgado et al., 2020). Opposing Fgf signals from the apical ectodermal ridge also influence the distribution of RA (Rosello-Diez et al., 2014) (Delgado et al., 2020). In this model, high Meis1 levels are associated with stylopod specification (Hoxa10, red; Figure 7); low Meis1, zeugopod specification (Hoxa11, green; Figure 7); and absent Meis1, autopod specification (Hoxa13, blue; Figure 7). Quail, chick, and turkey embryos have similar trunk widths; therefore, our data indicate that this parameter does not influence the distribution of RA in the wing bud. Instead, we implicated the RA-degrading enzyme Cyb26b1 in timing the removal of RA in the wing bud and in setting the pace of 5′ *Hox* gene activation. Thus, *Cyp26b1* expression in the quail wing bud increases at a significantly faster rate compared to the chick. In addition, the earlier depletion of RA signaling in the quail wing bud is indicated by the quicker loss of the distal domain of *Meis1* expression. The distribution of RA in the early wing bud could also be influenced by growth, although there is no clear relationship between species’ limb size at early stages. Therefore, species-specific rates of RA degradation are associated with the pace of 5′*Hox* gene expression and proximodistal specification.

Following the variable period of stylopod and zeugopod specification in avian wings (red, green), the autonomously timed program of distal development continues for a similar duration until patterning is complete (blue and white, Figure 7). The autonomous program is triggered by the depletion of RA (Rosello-Diez et al., 2014; Saiz-Lopez et al., 2015; Delgado et al., 2020) and coordinates the timing of autopod specification (blue, Figure 7), proliferation, differentiation, apoptosis, and organizer duration (white, Figure 7). However, since the autonomous program (autopod specification) is not linked to the incremental rate of growth that is comparable between species, this influences the size of the avian wing skeletal pattern (Figure 7), a 1.75-fold difference in length between the quail and turkey wings at HH29. These observations support the idea that organ size is largely intrinsically determined during embryogenesis as shown by classical experiments in which limb buds were exchanged between small and large species of salamander (Twitty and Schwind, 1931).

We previously demonstrated that cell proliferation in the early wing bud is at its highest rate during the period when RA signaling is active. However, once RA is removed from the wing bud, the autonomous program is activated and its duration is determined by the Bmp-dependent decline in proliferation rates in the distal mesoderm (Pickering et al., 2018). One possibility is that, upon being activated following the removal of RA signaling, Hoxa13/d13 influence cell proliferation via their regulation of *Bmp2/7* expression (Knosp et al., 2004). Although the removal of RA is required to start the autonomous program, it is unclear how this is achieved and it appears to require another undefined process. Therefore, it is likely that changes in the rate of cell proliferation in the limb are primarily governed by changes in repressive rather than inductive factors. We speculate that the cell cycle constitutes an overarching developmental timing mechanism, because it is intimately coupled to differentiation and apoptosis. Evidence that the cell cycle could constitute a developmental timer arises from the similarities between the chick wing and cultured oligodendroctye progenitor cells. In both cases, RA is implicated in triggering the onset of a cell cycle timer that involves the progressive lengthening of the G1 phase of the cell cycle (Gao et al., 1997, 1998; Durand and Raff, 2000; Chinnaiya et al., 2014), and is associated with the activation of D-cyclin-dependent kinase inhibitors, which are important negative regulators of the G1-S phase transition (Durand et al., 1998; Pickering et al., 2019). Unexpectedly, we revealed that the mesodermal cell proliferation rate does not correlate well with changes in growth between different species once the autonomous program has been activated. This finding fits with numerous observations in which the manipulation of proliferation failed to affect overall organ size (Conlon and Raff, 1999; Day and Lawrence, 2000). These considerations could suggest that the growth of avian wings is controlled by a global mechanism, such as metabolism/nutrition, which regulates the insulin/insulin-like-1 growth factor (IGF/IGF-like-1) mammalian target of rapamycin (mTOR) axis (Tumaneng et al., 2012). Consistent with this hypothesis, components of this pathway are expressed in chick wing buds (McQueeney and Dealy, 2001; Allan et al., 2003), and *in vitro* studies have implicated IGF in promoting limb outgrowth (Sears et al., 2012; Dealy and Kosher, 1996).

The widespread distribution of RA in the embryo could suggest that it plays a general role in developmental timing. Its coordinated depletion in left- and right-hand limb buds could ensure that they attain the same size. Furtheremore, RA promotes the expression of anterior *Hoxb* genes along the main body axis and is removed by Cyp26b1 to permit the expression of posterior *Hoxb* genes (Matsubara et al., 2017; Moreau et al., 2019). Thus, the relative timing of *Hoxb* expression is suggested to underlie evolutionary changes in avian limb position (Matsubara et al., 2017; Moreau et al., 2019). However, it remains to be determined whether RA affects developmental timing independently of growth along the main body axis, as we suggest that it does in the limb. Nevertheless, these considerations could suggest that RA coordinates developmental timing throughout the embryo.

### Limitations of the study

Although we have shown that prolonging RA signaling in the early wing bud can allow small species to develop with the timing of larger species, we have been unable to reverse timing in the opposite direction. Thus, another undefined mechanism ensures that *Hoxa13* expression and the autonomous distal program are activated at the correct time when RA signaling is blocked. We have been able to make large species develop with the timing of smaller species by performing grafts into a RA-rich environment, but this does not exclude the possibility that other unknown factors operate in parallel. Future work should be directed at deciphering the underlying mechanism of how the autonomous distal program is activated.

## STAR★Methods

### Key resources table

**Table:** 

REAGENT or RESOURCE	SOURCE	IDENTIFIER
**Antibodies**		

Digoxygenin-AP	Roche	11093274910

**Chemicals, peptides, and recombinant proteins**		

TTNPB	Sigma	71441-28
AGN193109	Sigma	171746-21-7
Lysotracker	Invitrogen	L-7528
TRIzol Reagent	Invitrogen	15596026

**Critical commercial assays**		

SuperScript III Reverse Transcriptase	Invitrogen	12574026
Direct-zol RNA kit	Zymo	R2061
SYBR Green Master Mix	Thermo Fisher	A46012
AGX1-2 beads (150 or 200 μm in diameter)	Sigma	Discontinued

**Deposited data**		

Flow cytometry source data	Mendeley	https://data.mendeley.com/datasets/25kj67jnnx/1

**Experimental models: Organisms/strains**		

*Gallus gallus domesticus*	Henry Stewart - UK	N/A
*Coturnix japonica*	Moonridge farm - UK	N/A
*Meleagris gallopavo domesticus*	Avara Foods Ltd - UK	N/A

**Oligonucleotides**		

Cyp26b1 (Forward) CCTGCAAG CTACCAATCCCT	Thermo Fisher	N/A
Cyp26b1 (Reverse) TTGCCGTA CTTCTCCCGTC	Thermo Fisher	N/A
18S rRNA (Forward) GTAACCCG TTGAACCCCATT	Thermo Fisher	N/A
18S rRNA (Reverse) CCATCCAA TCGGTAGTAGCG	Thermo Fisher	N/A

**Recombinant DNA**		

Chick Shh plasmid	Cheryll Tickle University of Bath	N/A
Chick Fgf8 plasmid	Cheryll Tickle University of Bath	N/A
Chick Hoxa11 plasmid	Cheryll Tickle University of Bath	N/A
Chick Hoxa13 plasmid	Cheryll Tickle University of Bath	N/A
Chick Hoxd13 plasmid	Cheryll Tickle University of Bath	N/A
Chick Meis1 plasmid	Cheryll Tickle University of Bath	N/A
Chick Sox9 plasmid	Cheryll Tickle University of Bath	N/A

### Resource availability

#### Lead contact

Further information and requests for resources and reagents should be directed to and will be fulfilled by the lead contact, Matthew Towers. (m.towers@sheffield.ac.uk).

#### Materials availability

This study did not generate new unique reagents.

### Experimental model and subject details

Bovans Brown chicken eggs (*Gallus gallus domesticus*), Japanese quail eggs (*Coturnix japonica*), and Bronze turkey eggs (*Meleagris gallopavo domesticus*) were incubated at 37°C and the embryos staged according to the Hamburger Hamilton system (Hamburger and Hamilton, 1951)) based on the number of somites present, and by characteristic morphological features of the wing bud. HH18/19 is reached by incubation day 3 in quails and chicks, and day 4 in turkeys, and is referred to in this study as 0 hours of wing outgrowth.

### Method details

#### Embryo measurements

Embryos of the appropriate age were dissected in PBS and measurements of the proximodistal axis were taken down the centre of the limb bud from the proximal boundary of the limb with the body wall, to the distal tip of the limb bud, accounting for elbow bend where appropriate. Embryo widths were measured between the wing buds in line with the body wall, and lengths, from the metencephalon to the tip of the tail (curved lines were measured using the transform command in Adobe Photoshop).

#### Whole mount *in situ* hybridisation

Embryos were fixed in 4% PFA overnight at 4°C then dehydrated in methanol overnight at −20°C. Embryos were then rehydrated through a methanol/PBS series, washed in PBS, then treated with proteinase K for 20 mins (10 μg/ml^−1^), washed in PBS, fixed for 30 mins in 4% PFA at room temperature and then prehybridised at 67°C for 2 hours (50% formamide/50% 2x SSC). 1 μg of antisense DIG-labelled mRNA probes were added to 1 ml of hybridisation buffer (50% formamide/50% 2x SSC) at 67°C overnight. Embryos were washed twice in hybridisation buffer, twice in 50:50 hybridisation buffer and MAB buffer, and then twice in MAB buffer, before being transferred to blocking buffer (2% blocking reagent 20% foetal bovine serum in MAB buffer) for 3 hours at room temperature. Embryos were transferred to blocking buffer containing anti-digoxigenin antibody (1:2000) at 4°C overnight, then washed in MAB buffer overnight before being transferred to NTM buffer containing NBT/BCIP and mRNA distribution visualised using a LeicaMZ16F microscope. Chick riboprobes were used to detect quail and turkey mRNA expression in all cases.

#### Flow cytometry

Polarizing regions or a 200μm^2^ block distal mesenchyme pooled from 8-12 replicate experiments were dissected in PBS under a LeicaMZ16F microscope using fine surgical scissors, and digested into single cell suspensions with trypsin (0.05%, Gibco) for 30 mins at room temperature. Cells were briefly washed in PBS, fixed in 70% ethanol overnight, washed in PBS and re-suspended in PBS containing 0.1% Triton X-100, 50 μg/ml^−1^ of propidium iodide and 50 μg/ml^−1^ of RNase A (Sigma). Dissociated cells were left at room temperature for 20 mins, cell aggregates were removed by filtration and single cells analysed for DNA content with a FACSCalibur flow cytometer and FlowJo software (Tree star Inc.). Based on ploidy values cells were assigned G1, S, or G2/M phases, and this was expressed as a percentage of the total cell number (5,000–12,000 cells in each case). Statistical significance of numbers of cells in different phases of the cell cycle (G1 vs. S, G2 and M) between pools of dissected wing bud polarizing region tissue (12–15 in each pool) was determined by Pearson’s χ^2^ tests to obtain two-tailed p values (significantly different being a *p-value* of less than 0.05 – as in (Chinnaiya et al., 2014). For the cell size analyses, chick and quail embryos were collected at the 12-hour time point and a 200μm^2^ block of distal mesodermal tissue was removed from the distal wing tip. Tissue from 6-10 embryos was pooled for each repeat of the experiment and then disaggregated in 0.05% trypsin (Sigma) for 30 mins. The disaggregated cells were then washed in PSB and analysed using a FACSCalibur flow cytometer. Doublet cells were used as a positive control reference during flow cytometric analyses. Quail and chick wing cell size was compared using the FSC (forward scatter - a measure of cell size as it quantifies how light is diffracted around the diameter of a single cell in suspension measuring the average fluorescence in arbitrary units (Collier, 2000).

#### Apoptosis analysis

Whole chick and quail wing buds were dissected in PBS and transferred to Lysotracker (Life Technologies, L-7528) PBS solution (1:1000) in the dark pre-warmed to 37°C. Wing buds were incubated for 1 hour at 37°C, washed in PBS, and fixed overnight in 4% PFA at 4°C. Wing buds were then washed in PBS and progressively dehydrated through a methanol series.

#### Polarizing region grafts

Polarizing region grafts were performed as described in (Stainton and Towers, 2018). Briefly, donor embryos were dissected in PBS and the polarizing regions removed using sharpened tungsten needles then transferred to the host embryo where they were grafted to equivalently sized regions of the host anterior limb bud and held in place with platinum pins of 25μm in diameter.

#### Quantitative PCR (qPCR)

Ten whole limb buds at 0, 6, 12 and 24 hours were dissected from either quail or chick embryos. Total RNA was extracted using TRIzol™ Reagent (Life Technologies), purified using a Direct-zol RNA kit (Zymo Research) and cDNA prepared using SuperScript III Reverse Transcriptase (Invitrogen). qPCR was performed on an Applied Biosystems StepOne RT-PCR machine using SYBR Green Master Mix (Thermo Fisher Scientific) and a primer set for *Cyp26b1* was designed against a sequence which was present in both chicken and quails, spanning exon junctions (Thermo Fisher Scientific). 5 ng cDNA was used per reaction (20μl volume) with cycle conditions of 95 °C for 20 sec, followed by 32 cycles of 95 °C for 1 sec and 60 °C for 20 sec. All reactions were carried out in triplicate and average C_T_ values normalized against eukaryotic *18S rRNA* endogenous control expression (Thermo Fisher Scientific).

#### Bead implantation

Sieved AGX1-2 beads (150 or 200 μm in diameter, Sigma) were soaked in a stable form of all-*trans*-retinoic acid, TTNPB (Sigma, 0.05 mg/ml dissolved in DMSO, Sigma) or AGN193109 (Sigma, 1 mg/ml dissolved in DMSO, Sigma) for 1 hour and then washed in DMEM before being grafted to the middle of wing buds using a sharp tungsten needle. TTNPB has been shown to diffuse from AGX1-2 beads over an approximate 12-20-hour period and can be used to model RA distribution in chick wing buds due to comparable patterning effects, kinetics and diffusion constants ((Eichele et al., 1984), (Eichele et al., 1985), (Eichele and Thaller, 1987).

### Quantification and statistical analysis

For flow cytometric analysis, statistical significance of numbers of cells in different phases of the cell cycle (G1 vs. S, G2 and M) between pools of dissected wing bud tissue (12–15 in each pool) was determined by Pearson’s χ^2^ tests to obtain two-tailed *p-value*s). For cell size analysis, differences between samples was determined by Student’s t-tests to obtain two-tailed *p-value*s. For quantitative PCR, unpaired Student’s *t* tests compared the mean relative expression and the significance of expression changes between appropriate samples to obtain two-tailed *p-value*s was determined. Applied Biosystems StepOne Software V2.3 was used to analyse the data. For embryo measurements, unpaired Student’s *t* tests or Wilcoxon tests were used to obtain to obtain two-tailed *p-value*s (see figure legends) between appropriate samples. GraphPad Prism8 was used to construct graphs. In all cases significantly different is taken as a *p-value* of less than 0.05 and GraphPad Prism8 was used to construct graphs.

## Data Availability

• All data reported in this paper will be shared by the lead contact upon request. • No code was generated in this paper. • Any additional information required to reanalyze the data in this paper is available from the lead contact upon request and the flow cytometry source data is available at Mendeley (https://data.mendeley.com/datasets/25kj67jnnx/1).

## Supplemental information

Document S1. Figures S1–S8

Document S2. Article plus supplemental information
