# Supplementary material for: Retinoic acid influences the timing and scaling of avian wing development
Source: Cell Rep. 2022 Jan 25;38(4):110288. doi: 10.1016/j.celrep.2021.110288 (PMC8810399; doi:10.1016/j.celrep.2021.110288)
Supplement: Document S1. Figures S1–S8 [file mmc1.pdf]

Cell Reports, Volume *38*

Supplemental information

Retinoic acid influences the timing and scaling of avian wing development

Holly Stainton and Matthew Towers

Supplementary information:

Retinoic acid influences the timing and scaling of avian wing development

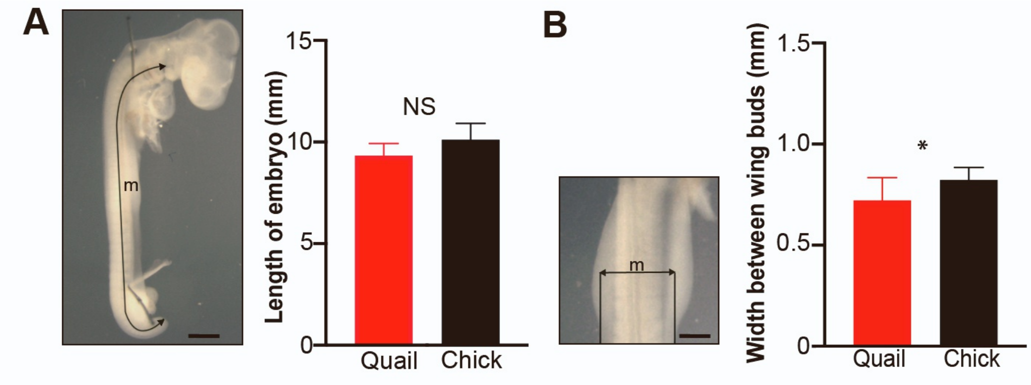

Figure S1, Related to Figure 1. Lengths and widths of quail and chick embryos at HH18/19

(A) Lengths of quail and chick embryos at 0h (HH18/19 – tail bud to metencephalon) are not significantly different, as indicated by Student's *t*-tests (*p*-value =0.055 *n*=6 and 10). **(B)** Widths of quail and chick embryos (between wing buds) at 0h are statistically different as indicated by Student's *t*-tests (*p*-value = 0.033, *n*=6 and 10). *p*-values: *=<0.05. Measurements (m) are shown on representative embryos. Scale bars: A = 500µm; B = 300µm

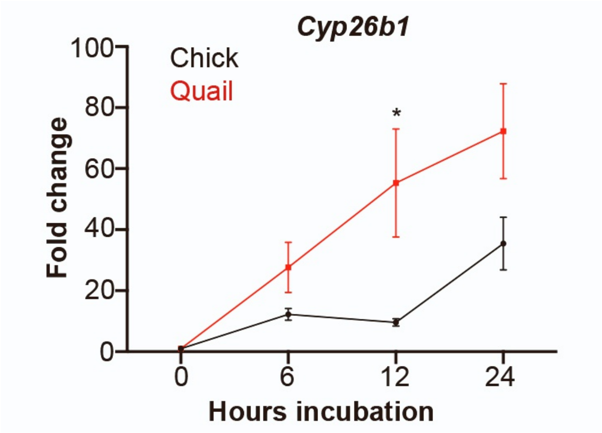

Figure S2, Related to Figure 3. Expression levels of *Cyp26b1* in quail and chick wing buds

A qPCR time-course reveals a significantly higher fold-change in *Cyp26b1* expression in 12h quail wing buds compared to chick wing buds (p-value=0.028) - the fold changes at 6 and 24h are not significantly different between species (p-value = 0.08 and 0.11, respectively). Student's unpaired t-tests were performed on n=3 (quail) and n=4 (chick) repeats of 10 pooled wing buds. p-values: *=<0.05

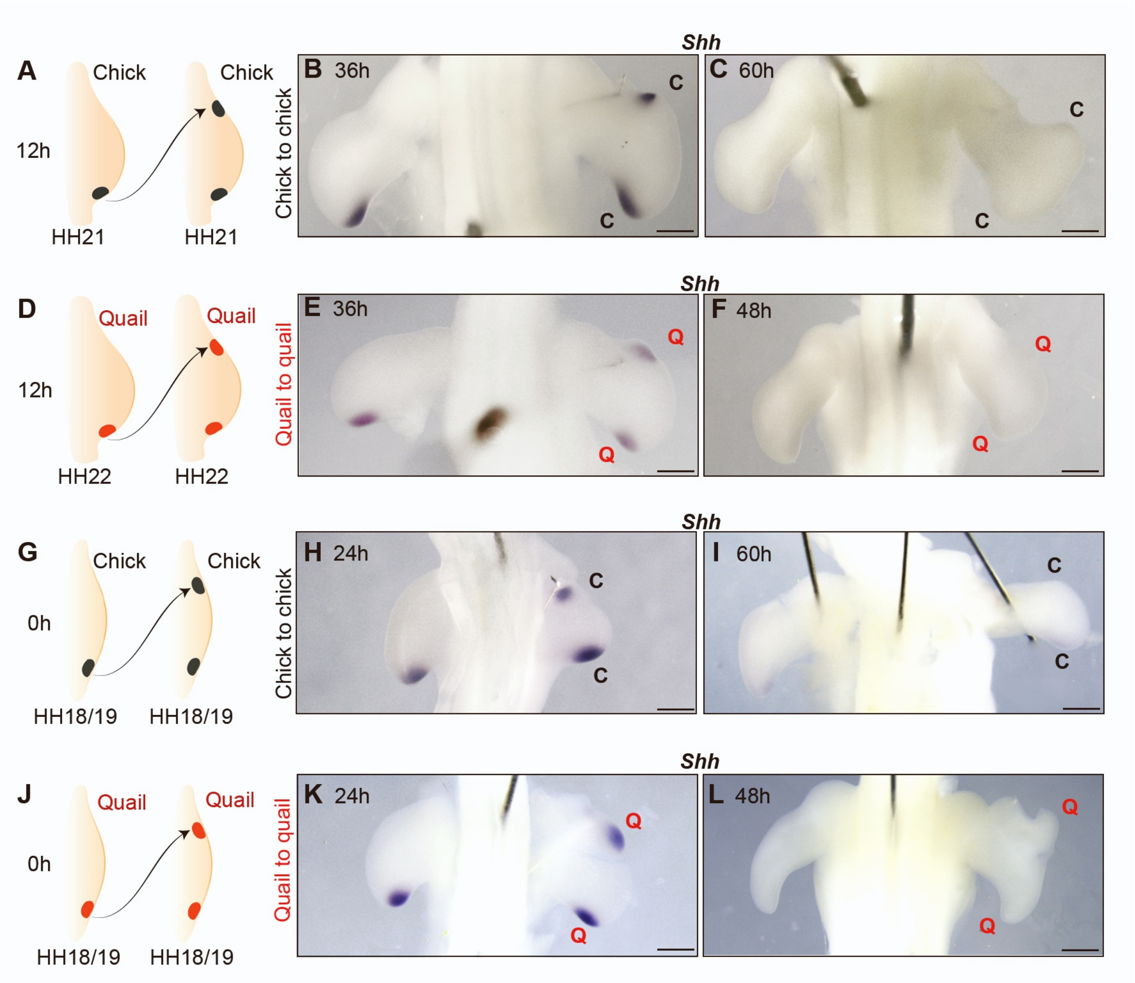

Figure S3, Related to Figure 4. *Shh* maintains its normal duration in intraspecies polarising region grafts

Control intraspecies chick (HH21) **(A-C)** and quail (HH22) **(D-F)** polarising region grafts made to the anterior margins of host wing buds at 12h. *Shh* is expressed at 36h **(B,** *n*=4/5, **e** *n*=6/6**)** and terminates at the correct time as shown at 60h **(C** *n*=4/4), and 48h **(F** *n*=5/6). Control intraspecies chick **(G-I)** and quail **(J-L)**, HH18/19, polarising region grafts made to the anterior margins of host wing buds at 0h. *Shh* is expressed at 36h **(H,** *n*=3/3, **K** *n*=4/4**)** and terminates at the correct time as shown at 60h **(I** *n*=3/4), and 48h **(L** *n*=3/3).

Scale bars: H, K = 300µm; F, B, E = 500µm C, I, L = 700µm

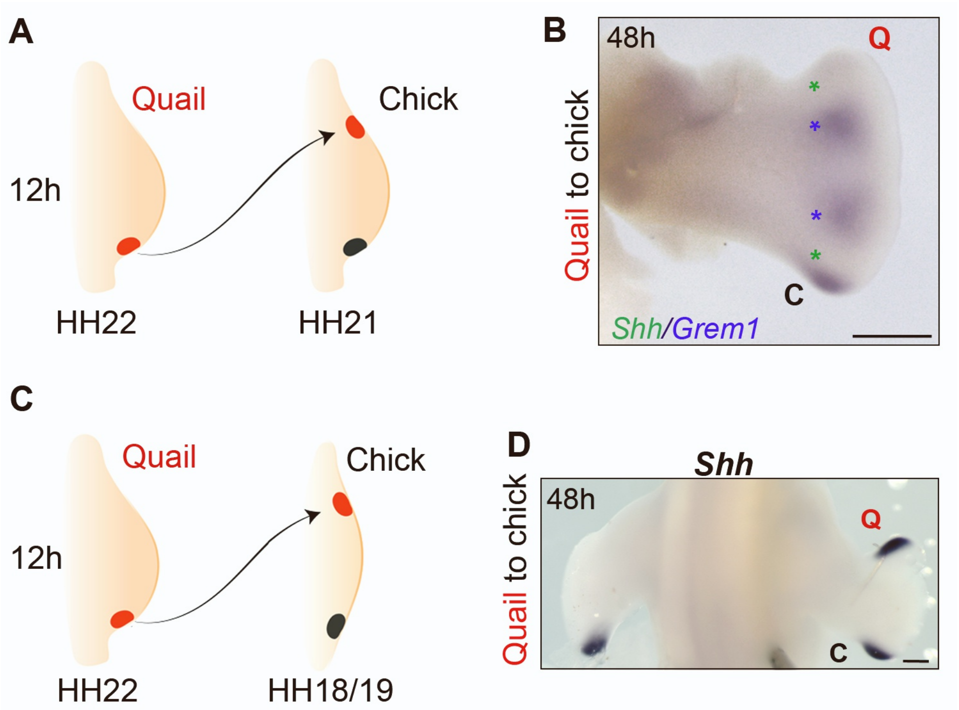

Figure S4, Related to Figure 4. *Shh* is intrinsically controlled by HH22 and can be reset in the early HH18/19 environment

(A) Polarising regions grafted from 12h quail wing buds (HH22) to the anterior margins of 12h chick wing buds (HH21). **(B)** Expression of *Shh* is terminated in the quail cells (upper green asterisk) and is observed in endogenous chick polarising region cells (lower green asterisk) at 48h. Both chick and grafted quail wing polarising regions induce a domain of *Grem1* expression in adjacent chick cells (purple asterisks): *Grem1* expression (lower purple asterisk) is adjacent to endogenous chick *Shh* expression, and a duplicated domain of *Grem1* expression is observed (upper purple asterisk) adjacent to where quail *Shh* would have been expressed. The loss of quail *Shh* expression demonstrates that *Grem1* expressing cells do not need to be displaced a critical distance by growth in order for *Shh* expression to be terminated at the correct time. Note equivalent distance between *Grem1* domains and anterior (403.3µm) and posterior (398.3µm) margins, *p*-value = 0.7944 indicated by Student's *t*-tests. (*n*= 3/3). **(C, D)** Polarising regions grafted from 12h quail wing buds (HH22) to the anterior margins of 0h chick wing buds (HH18/19) express *Shh* at 48h (*n*= 3/3).

Scale bar B = 600µm; D = 500µm

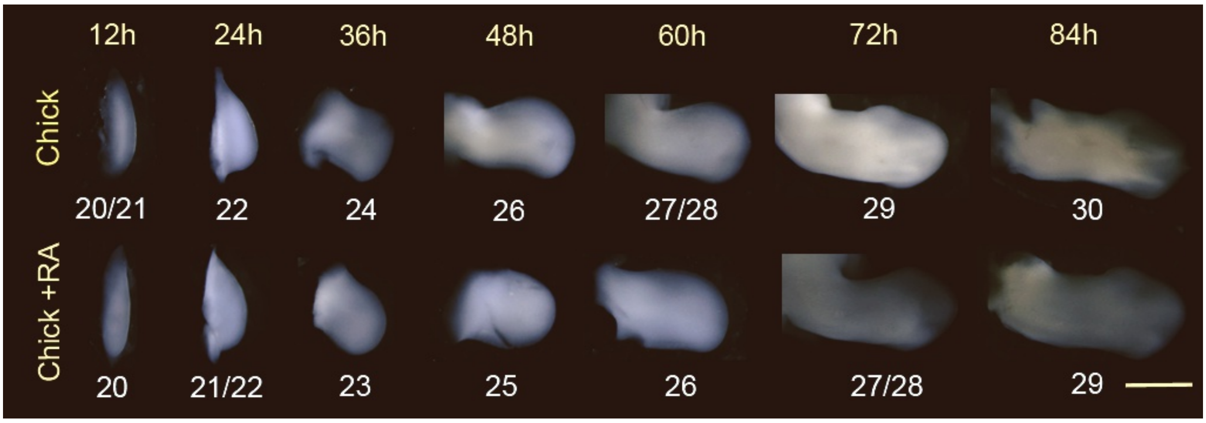

Figure S5, Related to Figure 5. Retinoic acid slows chick wing development

HH stages of chick wing buds treated with retinoic acid at 0h, compared with the contralateral untreated wing (*n*>6 for each stage). Note the control wing is flipped horizontally for comparison.

Scale bar: 1mm

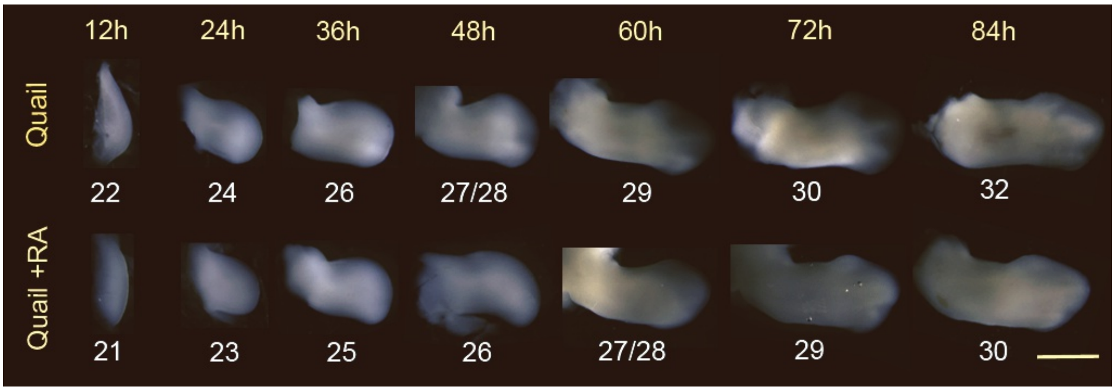

Figure S6, Related to Figure 5. Retinoic acid slows quail wing development

HH stages of quail wing buds treated with retinoic acid at 0h, compared with the contralateral untreated wing (n>6 for each stage). Note the control wing is flipped horizontally for comparison.

Scale bar: 1mm

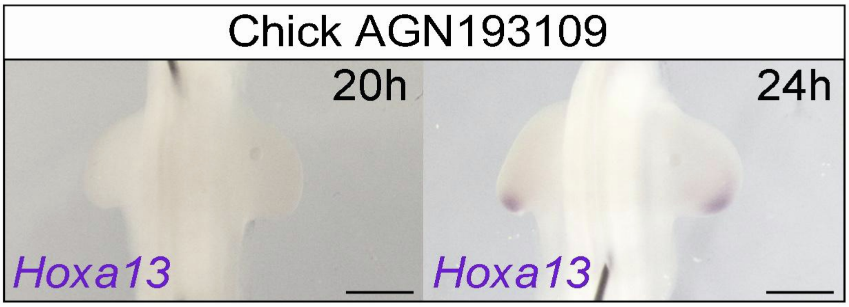

Figure S7, Related to Figure 5. Retinoic acid inhibition does not precociously activate *Hoxa13* expression

Right-hand chick wing buds were treated with AGN193109 at HH18 and HH19 and compared to control untreated left wings. Treatment at both stages (HH19 shown) does not affect the onset of *Hoxa13* expression at 24h (HH22) in both treated and untreated wings (*n*=6/6). Scale bar = 500µm

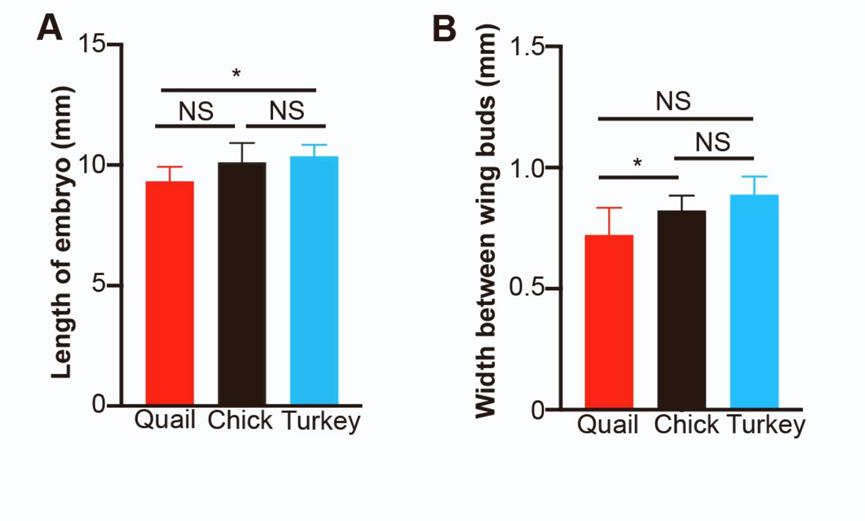

Figure S8, Related to Figure 6. Measurements of quail, chick and turkey embryos

(A) Lengths of turkey and chick embryos at 0h (HH18/19 – tail bud to metencephalon) are not significantly different (*p*-value =0.63), however turkeys are significantly longer than quail embryos (*p*-value=0.036), as indicated by Student's *t*-tests. **(B)** Width of turkey embryos (between wing buds) at 0h are not significantly different to chick embryos (*p*-value=0.140), or quail embryos (*p*-value=0.056), as indicated by Student's *t*-tests (*n*=3, 6 and 10 – turkey, quail, chick, respectively). *p*-values: *=<0.05
